# Effects of Childhood Emotional Abuse on Treatment Outcome in Adolescent Inpatients With Anorexia Nervosa

**DOI:** 10.1002/eat.24484

**Published:** 2025-06-06

**Authors:** Alessio Maria Monteleone, Marco Carfagno, Adrian Meule, Silke Naab, Giammarco Cascino, Ulrich Voderholzer, David R. Kolar

**Affiliations:** ^1^ Department of Psychiatry University of Campania Luigi Vanvitelli Naples Italy; ^2^ Department of Psychology University of Regensburg Regensburg Germany; ^3^ Schoen Clinic Roseneck Prien am Chiemsee Germany; ^4^ Department of Medicine, Surgery and Dentistry ‘Scuola Medica Salernitana’, Section of Neurosciences University of Salerno Salerno Italy; ^5^ Department of Psychiatry and Psychotherapy LMU University Hospital, LMU Munich Germany

**Keywords:** anorexia nervosa, emotional abuse, outcome, psychopathology, treatment

## Abstract

**Objective:**

Although childhood maltreatment, especially emotional abuse, is strongly linked to the psychopathology of anorexia nervosa (AN), the impact of such a traumatic experience on treatment outcome is not clear. This study aimed to explore how emotional abuse affects change in psychopathology during treatment.

**Method:**

Adolescents with AN (*n* = 331) completed the Childhood Trauma Questionnaire at admission to inpatient treatment and the Eating Disorder Inventory‐2, Patient Health Questionnaire‐9, Patient Health Questionnaire‐15, and Generalized Anxiety Disorder‐7 both at admission and at discharge. Relationships of emotional abuse with body mass index (BMI) and questionnaire scores at admission and at discharge were examined with percentage bend correlation coefficients. Changes in BMI and questionnaire scores from admission to discharge and whether these changes were moderated by emotional abuse were tested with robust mixed models.

**Results:**

Higher emotional abuse scores related to higher eating disorder, depressive, anxiety, and somatic symptoms but not to BMI at admission and at discharge. BMI increased and eating disorder, depressive, anxiety, and somatic symptoms decreased from admission to discharge but these changes were not moderated by emotional abuse scores.

**Discussion:**

Emotional abuse did not affect treatment response during hospitalization for AN, but it was associated with heightened eating and general psychological symptom severity at both hospital admission and discharge. Clinicians are advised to investigate a history of emotional abuse in adolescents with AN and to consider emotional abuse not as a predictor of treatment resistance, but as a psychological scar that persists regardless of symptom severity.

**Summary**

- The severity of emotional abuse is associated with that of psychopathology before and after treatment.
- Treatment induced change of eating, anxious, depressive, and somatic symptoms does not differ according to the severity of early emotional abuse.
- Emotional abuse does not predict treatment resistance in adolescents with Anorexia Nervosa.

## 1 Introduction

Eating disorders (EDs) are severe psychiatric disorders with modest rates of recovery and a high risk of relapse and hospitalization (Solmi et al. 2024). Treatment failure is often attributed to a lack of interventions focused on mechanisms that promote or maintain the illness (Monteleone and Abbate‐Daga 2024). Across these factors, childhood trauma is strongly linked to the etiopathogenesis of EDs (Solmi et al. 2020).

Individuals with EDs and a history of childhood maltreatment are characterized by a more severe form of the illness spanning heightened symptom severity, early age at onset, and more severe psychiatric comorbidity (Molendijk et al. 2017). Although higher rates of physical and sexual abuse are reported by individuals with EDs and binge/purge behaviors, the role of emotional abuse has recently emerged as the common pathway promoting the association between any type of childhood maltreatment and ED psychopathology (Monteleone, Cascino, et al. 2019; Monteleone et al. 2022) and psychiatric comorbidity (Guillaume et al. 2016). Emotional maltreatment is also included in the association between childhood maltreatment and quality of life in adolescents with EDs (Monteleone et al. 2023) or other psychiatric disorders (Kolar et al. 2024) before and after treatment.

Several studies have explored the putative psychological mechanisms mediating the connection between emotional maltreatment and ED symptoms, identifying a possible role of self‐esteem, alexithymia, and interpersonal problems (Barone et al. 2024; Rabito‐Alcón et al. 2021). Childhood trauma may also exert biological effects that have been observed in individuals with EDs (Cascino and Monteleone 2023; Lagan et al. 2024; Wiss et al. 2021) and contribute to the definition of a maltreated ecophenotype, as acknowledged in other psychiatric disorders (Rossi, Cassioli, Dani, et al. 2024). According to this assumption (Teicher and Samson 2013), more tailored treatments should be provided to patients with childhood maltreatment.

Childhood maltreatment may provide a psychological vulnerability promoting treatment resistance or risk of relapse in all psychiatric disorders: difficulties in developing a good therapeutic alliance, emotion regulation problems, and biological signatures (i.e., functional and structural brain differences) may mediate childhood maltreatment effects on treatment outcome (Teicher et al. 2022; Teicher and Samson 2013). However, it is not clear how and whether childhood maltreatment exerts its effects on treatment outcome in EDs. This gap has been clearly shown in two recent systematic reviews (Convertino and Mendoza 2023; Day et al. 2024), which have outlined the paucity of studies related to this issue in persons with EDs. Some studies failed to identify differences at the end of treatment between individuals with and without childhood maltreatment in terms of ED symptoms severity, whereas more severe symptomatology and higher rates of hospitalization and relapse were found at follow‐up in other studies (Anderson et al. 1997; Carter et al. 2012; Castellini et al. 2018). Instead, higher drop‐out rates in persons with EDs and childhood maltreatment consistently emerged among studies. Remarkably, both reviews concluded that inconsistencies in the study findings are likely attributable to the poor quality of the included studies and to the heterogeneity of the investigative methods used. Indeed, different types of traumas (namely, physical, sexual, and emotional trauma), and mixed ED diagnoses, participants' ages, and treatment settings have been involved in the assessment of childhood maltreatment effects on treatment outcome with insufficient data to enable comparisons by these conditions.

Therefore, this study aimed to address these literature gaps by investigating the effects of emotional abuse on the change in eating and general psychopathology between admission and discharge from a specialized inpatient unit in adolescents with anorexia nervosa (AN).

## 2 Method

### 2.1 Sample Characteristics

A secondary analysis from a dataset of adolescents with mental disorders admitted to the Schoen Clinic Roseneck Prien am Chiemsee, Germany, in the period between August 2015 and May 2021 (Kolar et al. 2024) was conducted. Adolescents with AN and atypical AN were included in the present sample. The current diagnosis was performed by physicians and psychologists with experience in child and adolescent psychiatry through the International Statistical Classification of Diseases, 10th revision criteria (World Health Organization 2004). All patients received individual and group‐delivered cognitive behavioral psychotherapy sessions with additional treatment components (e.g., exercise therapy, body image exposure). However, the presence of early trauma was not considered in adjusting the therapeutic modalities. Following the standard evaluation process, adolescents were asked to complete self‐report questionnaires at admission and discharge. The study was approved by the institutional review board of the ethics committee of the medical faculty at Ludwig‐Maximilians‐University Munich, Germany (No. 21‐0606).

### 2.2 Measures

The German version of the Childhood Trauma Questionnaire (CTQ; Bernstein et al. 2003; Klinitzke et al. 2012) was administered to assess childhood maltreatment. The CTQ is a self‐report questionnaire consisting of 28 items that assess five types of childhood maltreatment experience: emotional abuse, emotional neglect, physical abuse, physical neglect, and sexual abuse. The CTQ emotional abuse subscale demonstrated strong reliability (*ω* = 0.86). In the present study, the occurrence of emotional abuse was evaluated either as a continuous variable or as a categorical variable (namely, none/low and moderate/severe), according to the standardized cutoff score (Klinitzke et al. 2012).

The German version of the eating disorder inventory‐2 (EDI‐2; Garner 1991; Thiel et al. 1997) was employed to assess eating disorder‐related psychopathology. It is a self‐report questionnaire including 91 items. In line with scoring instructions for the EDI‐3, EDI‐2 subscales drive for thinness, body dissatisfaction, and bulimia were combined into an eating disorder symptomatology score (Garner 2004), as we were predominantly interested in the effects of childhood maltreatment on disordered eating symptomatology. The McDonald's omega for this score was *ω* = 0.95 at admission and *ω* = 0.94 at discharge.

The Patient Health Questionnaire‐SADS screener (Kroenke et al. 2010) was used to explore general psychological symptoms. This is a self‐report questionnaire which includes: 9 items assessing depression (namely, the depressive symptom scale from the patient health questionnaire) (PHQ‐9; Spitzer et al. 1999); 7 items assessing anxiety disorders constituting the generalized anxiety disorder scale (GAD‐7; Spitzer et al. 2006); 15 items investigating somatic symptoms and constituting the somatic symptoms scale from the patient health questionnaire‐15 (PHQ‐15; Kroenke et al. 2002). At admission, the PHQ subscales showed excellent reliability, with *ω* = 0.85 for somatic symptoms, *ω* = 0.88 for depressive symptoms, and *ω* = 0.91 for anxious symptoms. At discharge, the PHQ somatic symptoms scale showed a slight decrease in reliability (*ω* = 0.83), while the PHQ depression and anxiety scales maintained excellent reliability, with *ω* = 0.91 and *ω* = 0.92, respectively.

### 2.3 Statistical Analysis

Analyses were conducted with R version 4.4.2 in RStudio version 2024.12.0 (R Core Team 2021). Descriptive statistics were computed with the summarytools package version 1.0.1. McDonald's omega (McDonald 2013) was computed as the internal consistency coefficient for all questionnaires with the psych package version 2.4.12 (cf. McNeish 2018). Robust percentage bend correlation coefficients for testing relationships of the CTQ emotional abuse subscale with BMI, questionnaire scores at admission and discharge, and length of stay were computed with the WRS2 package version 1.1‐6 (cf. Mair and Wilcox 2020).

Changes in BMI and questionnaire scores from admission to discharge were tested with robust mixed models with the robustlmm package version 3.3‐1 (Koller 2016). Specifically, separate models were calculated for each dependent variable that included a fixed effect of time (admission vs. discharge) and a random intercept. As robustlmm does not produce parameter‐specific *p* values, we used the workaround described by Geniole et al. (2019) and fitted non‐robust models with the lme4 package version 1.1‐36 (Bates et al. 2015) to obtain Satterthwaite‐approximated degrees of freedom. Two‐sided *p* values were then computed for the robust *t*‐values using the approximated degrees of freedom. Cohen's d was computed as effect size with the effectsize package version 1.0.0 and commonly applied thresholds for interpreting effects as small, medium, and large are 0.2, 0.5, and 0.8 (Cohen 2013).

In another set of models, we added the CTQ emotional abuse subscale and a time × emotional abuse interaction to examine whether emotional abuse scores moderated changes in BMI and questionnaire scores from admission to discharge. In additional models, we also added length of stay to examine whether controlling for this variable changed any effects of interest. To rule out that only severe forms of emotional abuse impact psychopathology, emotional abuse was also categorized in two groups (namely, none/low and moderate/severe) and additional mixed models were run including this resulting categorical variable. Because of the large sample size and numerous inferential tests, we considered effects as significant when *p* < 0.005 (Benjamin et al. 2018). The data and code with which results can be reproduced are available at https://osf.io/a7h25/.

## 3 Results

### 3.1 Sample Characteristics

The study sample was composed of 331 adolescents with AN. Most of the sample was female (98.5%), while only 1.5% identified as male. At the time of admission, participants had an average BMI of 15.12 kg/m^2^ (SD = 1.85). The average length of hospital stay was 104.85 days (SD = 50.85), with considerable variability among individuals. At admission 32.93% of the sample had AN (no subtype specified), 13.6% had binge/purge subtype, 45.02% had restricting subtype, and 8.46% had atypical AN. Psychiatric comorbidities were highly prevalent, affecting nearly two‐thirds of the sample (67.67%). 61.6% of participants met the criteria for affective disorders, while 21.45% had a comorbid anxiety disorder. According to the standardized cut‐off score, 14.5% (*n* = 48) of participants experienced moderate/severe levels of emotional abuse, while the remaining 85.5% (*n* = 283) reported none/low levels of emotional abuse.

### 3.2 Robust Correlation Analysis

The results of Robust Correlation Analysis exploring the association between emotional abuse and clinical variables are reported in Table 1.

**TABLE 1:** Results of robust correlation analysis assessing the association between childhood emotional abuse and BMI and symptom scores at admission to discharge.

	Admission *r* (*p*)	Discharge *r* (*p*)
BMI	0.031 (0.574)	0.081 (0.136)
EDI‐2 eating disorder symptomatology	0.254 (< 0.001)	0.172 (0.017)
PHQ somatic symptoms	0.321 (< 0.001)	0.206 (< 0.001)
PHQ depressive symptoms	0.345 (< 0.001)	0.258 (< 0.001)
PHQ anxious symptoms	0.351 (< 0.001)	0.26 (< 0.001)

Abbreviations: BMI, body mass index; EDI‐2, Eating Disorder Inventory‐2; PHQ, patient health questionnaire.

Childhood emotional abuse was positively associated with eating disorder symptomatology severity at admission and at discharge. No significant correlation was found between emotional abuse and BMI at either admission or discharge. At admission, childhood emotional abuse was strongly correlated with somatic, depressive, and anxious symptoms. These correlations remained significant at discharge. Childhood emotional abuse was not correlated with length of stay (*r*_pb_ = 0.05, *p* = 0.419).

### 3.3 Robust Mixed Models

The results of robust mixed models are reported in Table 2.

**TABLE 2:** Results of robust mixed models assessing whether childhood emotional abuse moderated changes in BMI and symptom scores from admission to discharge.

	Admission mean (SD)	Discharge mean (SD)	*β*	*p*	Effect size [95% CI]	Time × CEA (*p*)
BMI	15.12 (1.85)	17.84 (1.75)	2.74	< 0.001	−1.58 [−1.74, −1.41]	0.74
EDI‐2 eating disorder symptomatology	81.50 (21.18)	68.42 (20.67)	−12.68	< 0.001	0.73 [0.59, 0.87]	0.038
PHQ somatic symptoms	10.57 (5.52)	7.90 (4.55)	−2.39	< 0.001	0.48 [0.35, 0.61]	0.008
PHQ depressive symptoms	13.86 (6.25)	8.69 (5.65)	−5.00	< 0.001	0.61 [0.72, 1.01]	0.368
PHQ anxious symptoms	9.95 (5.30)	6.97 (4.77)	−2.86	< 0.001	0.87 [0.47, 0.74]	0.428

Abbreviations: BMI, body mass index; CEA, childhood emotional abuse; EDI‐2, eating disorder inventory‐2; PHQ, patient health questionnaire.

BMI increased from admission to discharge but with a statistically non‐significant interaction between childhood emotional abuse and time, indicating that emotional abuse did not impact BMI change over the treatment course. When treatment duration was included in the model, the interaction between childhood emotional abuse and time remained not significant (*p* = 0.763).

The eating disorder symptomatology significantly decreased from admission to discharge. The interaction between time and emotional abuse was not statistically significant at a conservative threshold of *p* < 0.005, even when controlling for treatment duration (*p* = 0.037). Similarly, a significant reduction in PHQ somatic symptoms was observed from admission to discharge. The interaction between time and emotional abuse was not statistically significant at a conservative threshold of *p* < 0.005, even when controlling for treatment duration (*p* = 0.037). Similar decreases in PHQ depressive and anxiety symptoms over time were found for depressive and anxiety symptoms. In both models, childhood emotional abuse did not significantly moderate the change in symptoms over time, even when controlling for treatment duration (*ps* > 0.324).

When emotional abuse was included in the robust mixed models as a categorical variable, emotional abuse did not significantly moderate the change of symptoms over the course of treatment (see Supporting Information).

## 4 Discussion

This study investigated the effects of childhood emotional abuse on treatment outcome in a sample of hospitalized adolescents with AN. Eating, anxious, somatic, and depressive symptoms improved at the end of treatment without differences between those with and without emotional abuse, but the severity of early emotional abuse was associated with that of symptomatology at both baseline and end of treatment.

These findings add evidence to the association between emotional abuse and psychopathology, but not BMI, in individuals with AN (Caslini et al. 2016; Monteleone, Cascino, et al. 2019) showing that the effects of emotional abuse are still evident at the end of treatment, at least for a subset of symptoms. Our results expand previous findings, as most previous studies exploring the effect of childhood maltreatment on treatment outcome reported childhood sexual abuse as the type of trauma with an effect on the trajectory of eating pathology over time (Eielsen et al. 2024; Ridley 2009; Vrabel et al. 2010). However, these studies included samples with different ED diagnoses and mixed age (Convertino and Mendoza 2023; Day et al. 2024), while the current findings come from a more homogenous population consisting of adolescents with restrictive eating behaviors. The lack of association between emotional abuse and BMI is not unexpected, considering the growing body of evidence indicating that BMI is a marker of mortality risk (Solmi et al. 2024) rather than psychopathology severity in AN (Gorrell et al. 2019; Monteleone et al. 2021; Smith et al. 2017). The severity of undernutrition in AN has been recognized as being more strongly associated with factors like individual weight history, rather than BMI alone (Coners et al. 1999; Peebles et al. 2010).

To our knowledge, this is the first study investigating the impact of childhood emotional abuse on treatment outcome in terms of both eating specific symptoms and general psychopathological symptoms. This is highly relevant considering a growing body of research describing affective and somatic symptoms as part of the psychopathological core of AN (Monteleone, Mereu, et al. 2019; Oldershaw et al. 2019). Therefore, the persistence of heightened anxious, depressive, and somatic symptoms in adolescents with more severe emotional abuse experiences suggests a residual vulnerability to psychopathology, even after clinical improvement or remission of AN. Given the high drop‐out rates observed in persons with EDs and childhood maltreatment (Convertino and Mendoza 2023; Day et al. 2024), investigating a potential association between early emotional abuse and early treatment discontinuation would have been of considerable interest. This hypothesis was not supported by correlation analyses, which did not show a relationship between emotional abuse and duration of hospitalization. However, a direct assessment of discharge reason would have better clarified the role of emotional abuse in treatment dropout.

Remarkably, in the present study, adolescents with AN reported no difference in symptom change during treatment regardless of the severity of childhood emotional abuse. This finding is partially novel in the literature: only one study (Billman Miller et al. 2024) explored the effects of childhood emotional abuse in a sample with AN and atypical AN and found that emotional abuse predicted less improvement in shape and weight overvaluation at discharge. However, this study was conducted in a small sample of adults with AN included in a partial hospitalization program. On the other hand, most previous studies have explored the effects of non‐specific childhood traumatic events and/or have been conducted in samples with mixed ED diagnoses (Convertino and Mendoza 2023; Day et al. 2024) and suggested that childhood trauma history did not moderate ED treatment outcomes (Liebman et al. 2025). Although the present findings are in line with this evidence, it is important to outline that higher emotional abuse was associated with more severe psychopathology both before and after treatment. This aligns with the *maltreated ecophenotype* theory (Teicher and Samson 2013), which claims that the effects of childhood maltreatment persist as a *scar* in the long term, even after treatment and regardless of the occurrence of symptoms defining a psychiatric diagnosis. From a clinical point of view, this means heightened vulnerability to the persistence of the illness or to subsequent episodes of relapse. According to the *maltreated ecophenotype* theory, treatments including additional interventions should be provided to individuals with psychiatric disorders and a childhood traumatic history. In this line, there is preliminary evidence of effectiveness for add‐on treatments (Rossi, Cassioli, Cecci, et al. 2024; van Beers et al. 2025). Although the addition of trauma‐focused interventions to evidence‐based treatments for EDs has been proposed (Brewerton 2019) and no adverse events have been reported, the current evidence is insufficient to recommend their routine use in individuals with EDs. Furthermore, it is important to outline that most of these treatments were originally designed for addressing post‐traumatic stress disorder and not childhood emotional abuse. Preliminary findings (Kopland et al. 2024; Rodgers et al. 2019) suggested that treatment‐induced changes may occur through different mechanisms in those with versus without childhood maltreatment. Qualitative findings outlined that individuals with EDs and childhood maltreatment may benefit from integrated interventions focusing on dysfunctional alliance processes (Olofsson et al. 2024). However, exploring mechanisms of treatment‐induced change is out of the scope of this study and will have to be explored by future research.

Limitations of the study need to be acknowledged. First, the study sample was composed of hospitalized adolescents mainly with AN restricting type: although avoiding heterogeneity in the study sample is recommended (Convertino and Mendoza 2023; Day et al. 2024), a limited generalizability of the present findings should be considered. Second, treatment effects were evaluated only at discharge without follow‐up assessment: this is an important aim for future research, as recovery trajectories may diverge after treatment between adolescents with and without childhood emotional abuse. Third, post‐traumatic stress symptoms are supposed to promote relapse in individuals recovered from EDs with childhood maltreatment (Convertino and Mendoza 2023), but they have not been evaluated in this study. Fourth, although the assessment was conducted by expert physicians and psychologists, the diagnosis was not standardized through a clinical interview. Fifth, the effects of other kinds of childhood traumatic events (namely, sexual and physical abuse) on treatment outcome have not been explored. Despite these limitations, the recruitment of a sample with a unique diagnosis and a restricted age range, the unique treatment setting, and the large sample size are strengths of this work. In addition, this is the first study in individuals with AN evaluating the effects of emotional abuse on treatment outcome measured not only in terms of eating symptoms but also of a wide range of general psychological symptoms.

## 5 Conclusions

This study supports the importance of investigating the severity of emotional abuse events in adolescents with AN: this is even more important considering that individuals with EDs who have experienced early trauma report that their trauma was often ignored and untreated by clinicians (Brewerton et al. 2019). The present findings suggest that adolescents with AN benefit from a treatment primarily designed to address their ED regardless of the severity of early emotional abuse: clinicians may consider the occurrence of emotional abuse not as a predictor of treatment resistance, but as a predictor of disorder severity at clinical admission and at discharge from hospitalization. Although adolescents with AN and high emotional abuse benefit from ED treatment for their disorder, early emotional abuse seems to provide a scar that persists regardless of symptom severity and may predict relapse, although this hypothesis deserves further investigation through follow‐up assessment.

## Author Contributions

**Alessio Maria Monteleone:** conceptualization, writing – original draft, writing – review and editing, methodology. **Marco Carfagno:** methodology, writing – review and editing, formal analysis. **Adrian Meule:** investigation, methodology, writing – review and editing, software, formal analysis. **Silke Naab:** investigation, writing – review and editing. **Giammarco Cascino:** conceptualization, writing – review and editing. **Ulrich Voderholzer:** investigation, supervision, writing – review and editing, data curation. **David R. Kolar:** writing – review and editing, methodology, software, formal analysis, supervision, data curation, investigation.

## Conflicts of Interest

The authors declare no conflicts of interest.

## Supporting information


Data S1.

## Data Availability

The data that support the findings of this study are openly available in OSF at https://osf.io/a7h25/.
