# Supplementary material for: Effects of Childhood Emotional Abuse on Treatment Outcome in Adolescent Inpatients With Anorexia Nervosa
Source: Int J Eat Disord. 2025 Jun 6;58(9):1769–76. doi: 10.1002/eat.24484 (PMC12423589; doi:10.1002/eat.24484)
Supplement: Supplementary file 1 — Data S1. [file EAT-58-1769-s001.docx]

*Robust mixed models with emotional abuse as a categorical variable*

Robust mixed models showed an increase in BMI from admission to discharge with a large effect size (β = 2.743, p < 0.001, d = -1.58, 95% CI [-1.74, -1.41]) but with a statistically non-significant interaction between childhood emotional abuse and time, indicating that emotional abuse did not impact BMI change over treatment course (p = 0.91). When treatment duration was included in the model the interaction between childhood emotional abuse and time remained not significant (p=.92).

The eating disorder symptomatology significantly decreased from admission to discharge (β = -12.685, p < 0.001; d = 0.73, 95% CI [0.59, 0.87]), also indicating a substantial reduction in eating disorder severity over the course of treatment. The interaction between time and emotional abuse was not statistically significant at a conservative threshold of p < .005 (β = -7.03; p = 0.023), even when controlling for treatment duration (p=.022). Similarly, a significant reduction in PHQ somatic symptoms was observed from admission to discharge (β = -2.390, p < 0.001; d = 0.48, 95% CI [0.35, 0.61]). The interaction between time and emotional abuse was not statistically significant at a conservative threshold of p < .005 (β = -0.214, p = 0.019), even when controlling for treatment duration (p=.018).

Similar decreases in PHQ depressive and anxiety symptoms over time were found (-5.00 ≤ β ≤ -2.86, ps < .001; 0.61 ≤ d ≤ 0.87, 95% CI for depressive [0.72, 1.01] and for anxiety symptoms 95% CI [0.47, 0.74]). In both models, childhood emotional abuse did not significantly moderate the change in symptoms over time (ps > .272), even when controlling for treatment duration (ps > .231).
